# Links among Obesity, Type 2 Diabetes Mellitus, and Osteoporosis: Bone as a Target

**DOI:** 10.3390/ijms25094827

**Published:** 2024-04-28

**Authors:** Monika Martiniakova, Roman Biro, Noemi Penzes, Anna Sarocka, Veronika Kovacova, Vladimira Mondockova, Radoslav Omelka

**Affiliations:** 1 Department of Zoology and Anthropology, Faculty of Natural Sciences and Informatics, Constantine the Philosopher University in Nitra, 949 01 Nitra, Slovakia; roman.biro@ukf.sk (R.B.); vkovacova@ukf.sk (V.K.); 2 Department of Botany and Genetics, Faculty of Natural Sciences and Informatics, Constantine the Philosopher University in Nitra, 949 01 Nitra, Slovakia; noemi.penzes@ukf.sk (N.P.); sarocka.anna@gmail.com (A.S.); vmondockova@ukf.sk (V.M.); romelka@ukf.sk (R.O.)

**Keywords:** obesity, type 2 diabetes mellitus, osteoporosis, adipose tissue, bone tissue, BMD, fracture risk, treatment

## Abstract

Obesity, type 2 diabetes mellitus (T2DM) and osteoporosis are serious diseases with an ever-increasing incidence that quite often coexist, especially in the elderly. Individuals with obesity and T2DM have impaired bone quality and an elevated risk of fragility fractures, despite higher and/or unchanged bone mineral density (BMD). The effect of obesity on fracture risk is site-specific, with reduced risk for several fractures (e.g., hip, pelvis, and wrist) and increased risk for others (e.g., humerus, ankle, upper leg, elbow, vertebrae, and rib). Patients with T2DM have a greater risk of hip, upper leg, foot, humerus, and total fractures. A chronic pro-inflammatory state, increased risk of falls, secondary complications, and pharmacotherapy can contribute to the pathophysiology of aforementioned fractures. Bisphosphonates and denosumab significantly reduced the risk of vertebral fractures in patients with both obesity and T2DM. Teriparatide significantly lowered non-vertebral fracture risk in T2DM subjects. It is important to recognize elevated fracture risk and osteoporosis in obese and T2DM patients, as they are currently considered low risk and tend to be underdiagnosed and undertreated. The implementation of better diagnostic tools, including trabecular bone score, lumbar spine BMD/body mass index (BMI) ratio, and microRNAs to predict bone fragility, could improve fracture prevention in this patient group.

## 1. Introduction

Obesity, type 2 diabetes mellitus (T2DM), and osteoporosis represent a currently growing health problem, as their prevalence is escalating and they are associated with increased morbidity, mortality, and health expenditures, mostly in older individuals. According to the World Health Organization (WHO), obesity is considered an excessive accumulation of fat that can have a detrimental effect on health. For the general population, a body mass index (BMI) greater than or equal to 30 kg/m^2^ is defined as obesity. However, for Asians, obesity is defined as a BMI greater than or equal to 25 kg/m^2^. It is predicted that by 2030, 20% of women, 14% of men, and more than 1 billion people will be living with obesity [1,2,3]. Obesity is the most important risk factor for T2DM, which is characterized by chronic hyperglycemia, insulin resistance, and inefficient insulin secretion and action. T2DM may be diagnosed based on plasma glucose criteria, either the fasting plasma glucose value (≥126 mg/dL; ≥7.0 mmol/L) or the 2 h plasma glucose value (≥200 mg/dL; ≥11.1 mmol/L) during an oral glucose tolerance test, and/or based on glycated hemoglobin (HbA1C) criteria (≥6.5%; 48 mmol/mol). The global prevalence of T2DM is expected to be 592 million by 2035 [4,5,6]. The WHO defined osteoporosis as a disease of low bone mass with bone mineral density (BMD) equal to or less than −2.5 standard deviations (SD) of the mean value for young, healthy people (a T-score ≤ − 2.5 SD). Disrupted bone microarchitecture and elevated risk of fragility fractures are typical for this disease. The worldwide prevalence of osteoporosis has been reported at 18.3%; in particular, the prevalence in Africa and Europe is much higher. More than 30 million people in Europe are expected to be affected by osteoporosis by 2050 [7,8,9].

Considering osteoporosis, gender must be taken into account. In women, primary osteoporosis is classified based on aging as either type 1 (postmenopausal osteoporosis) or type 2 (senile osteoporosis). Senile osteoporosis usually develops after the age of 70 and is determined at a ratio of 2:1 in women and men [10,11]. Men experience delayed bone loss compared to women due to a slower rate of decline in testosterone and estradiol. Therefore, fractures occur about 10 years later in men than in women [12]. Secondary osteoporosis results from secondary causes of bone loss, which can include various clinical and lifestyle factors. In men, secondary osteoporosis has a higher frequency. Two thirds of older men, over 50% of premenopausal women, and 30% of postmenopausal women are reported to suffer from secondary osteoporosis [13,14].

There is some overlap between factors influencing obesity, T2DM, and osteoporosis. Age-related bone loss, reduced hematopoiesis, and increased adipogenesis are considered hallmarks of aging and are associated with impaired bone quality and health [15]. However, metabolic disturbances in an organism, such as obesity and T2DM, can accelerate harmful alterations in bone homeostasis and contribute to the early onset of osteoporosis [9]. In addition, secondary causes of osteoporosis, including T2DM, glucocorticoids, and immobility, are related to bone marrow adiposity. Both bone remodeling and adiposity are regulated by the hypothalamus and the sympathetic nervous system. The cross-talk between adipose and bone tissues is mediated by adipocytes, osteoblast- and osteoclast-derived factors, and vitamin D. Ultimately, adipocytes and osteoblasts derive from a common progenitor [16,17].

In general, obesity and T2DM are linked to normal or higher BMD; however, paradoxically, both aforementioned conditions are associated with an elevated risk of fragility fractures at specific sites. In the case of obesity, this phenomenon is denoted by the term “obesity paradox” [1,2]. The aim of this review was to summarize the interactions among obesity, T2DM, and osteoporosis, considering bone as a target organ. Most scientific studies specifically address only the relationships between obesity and osteoporosis, and T2DM and osteoporosis. However, since almost all individuals with T2DM are obese (interactions between adipose and bone tissues are also relevant in individuals with T2DM) and increased bone fragility occurs in both obesity and T2DM, the links between these three diseases were presented in our review. Furthermore, in both obesity and T2DM, biologically active molecules responsible for impaired bone quality were characterized, as well as pharmacotherapy in connection with reducing the incidence of fragility fractures and also shortcomings in the diagnosis of bone damage. In this context, microRNAs have been described as promising prognostic biomarkers for predicting not only the occurrence of aforementioned diseases but also bone fragility. Therefore, such an overview is much more comprehensive and provides the most up-to-date knowledge in the given field. In connection with the aging of an increasingly obese population, understanding the links among obesity, T2DM, and osteoporotic fractures becomes an urgent need in order to reduce the societal and individual costs of treating these diseases and their secondary complications.

## 2. Interactions between Adipose Tissue and Bone Tissue

Current studies suggest that adipose tissue can negatively affect bone health, challenging the traditional paradigm that increased fat mass is beneficial for the skeleton. In general, adipose tissue is made up of adipocytes distributed in the organism, especially in the subcutaneous and visceral fat. The function of adipose tissue varies from organs as energy storage sites to endocrine organs secreting various factors that can influence different organ systems and regulate energy metabolism [3,18]. In the context of obesity and T2DM, intramuscular fat and bone marrow adipose tissue also play a significant role in skeletal homeostasis and bone fragility [4,9].

It should be noted that in connection with aging, there is a redistribution of adipose tissue with a decrease in subcutaneous fat and an increase in visceral fat, intramuscular fat, and bone marrow adipose tissue. It is associated with greater bone fragility, increased risk of falls and fractures, and it is similar to the redistribution caused by obesity. As a result, its harmful effects are elevated in elderly and obese patients [19].

The distribution of adipose tissue in specific regions, namely, subcutaneous and visceral fat, has been shown to be an excellent indicator of bone health compared to overall adiposity. It is known that subcutaneous and visceral fat share a common gene pool; however, their distinct structures and functions differ, leading to different physiological consequences [20,21]. For example, visceral adipocytes show increased insulin resistance compared to subcutaneous adipocytes, have a more active metabolism profile, and higher lipolysis toxicity [22]. Subcutaneous adipocytes show higher expression of adiponectin and leptin compared to visceral adipocytes [17,23]. Moreover, subcutaneous and visceral fat induce various inflammatory conditions [24]. In any case, visceral fat has been shown to be more detrimental to bone health [20,25]. The visceral reservoir releases adipocytokines that stimulate hepatic release of acute phase response proteins such as C-reactive protein (CRP) and are related to macrophages that secrete inflammatory cytokines including interleukin-6 (IL-6), tumor necrosis factor alpha (TNF-α), plasminogen activator inhibitor-1, and monocyte chemotactic protein-1 [26]. IL-6 can stimulate osteoclasts to elevate the rate of bone resorption, while higher levels of circulating CRP are linked to higher levels of N-terminal telopeptide of type 1 collagen (NTx), a marker of bone resorption and lower bone mass [27].

Most research studies pointed to a link between increased visceral fat and damaged bone structure. In a study by Bredella et al. [28], overweight and obese men with high visceral fat were shown to possess worse bone microarchitecture and mechanical properties versus those with low visceral fat. Kim et al. [29] revealed that femoral neck BMD was significantly reduced in individuals with metabolic syndrome. Waist circumference (WC, an indicator of visceral fat mass) was the most important component of this negative association, indicating that visceral fat may lead to bone loss. Similarly, in a study by Gilsanz et al. [30], visceral fat negatively correlated with most parameters of bone microstructure and with strength in adult women, suggesting that visceral fat serves as a unique pathogenic fat reservoir. Conversely, subcutaneous fat had a favorable effect on these bone parameters. A protective impact of subcutaneous fat on bone health (femoral neck BMD in elderly women) was also determined by Marquez et al. [31]. On the other hand, Wang et al. [32] failed to discern any detectable association between subcutaneous fat and lumbar spine BMD. Regarding visceral fat, a negative correlation between this fat and lumbar spine BMD was noted in both perimenopausal and postmenopausal women [33]. However, Liu et al. [34] found a positive association between visceral fat and BMD of the radius and tibia, although this association lost significance after adjustment for body weight or BMI. Zhang et al. [35] did not identify a significant correlation between visceral fat and lumbar spine BMD. According to Ng et al. [25], the relationship between visceral and subcutaneous fat depots and bone health is more complex and is specific for age, gender, menopausal status, bone compartment, and adipose depot. Lin et al. [18] revealed a negative association between subcutaneous fat and lumbar spine BMD. On the other hand, a non-linear (U-shaped) relationship between visceral fat and BMD was determined. Therefore, individuals exhibiting a certain range of visceral adiposity along with increased subcutaneous fat face an increased susceptibility to osteoporosis. Thus, research findings have supported the claim that the association between adipose tissue and bone tissue exhibits variation dependent on the specific type of fat. In a study by Hind et al. [36], visceral and total adiposity were significant predictors of prevalent vertebral fractures in postmenopausal women.

Overall, intramuscular fat content is elevated in obesity and may be linked to impaired muscle function and strength, which is termed “sarcopenic obesity”. In sarcopenic obesity, intramuscular lipids and their derivatives accumulate both inter- and intra-myocellularly and induce mitochondrial dysfunction, reduced mitochondrial mass, impaired energy production, and increased oxidative stress [37,38]. Poor muscle function could lead to falls, fall injuries, and fractures, and there is evidence to suggest an excess of falls in obese people [4,39]. Therefore, intramuscular fat can serve as an independent predictor of falls and subsequent fractures in older adults [40,41]. Lang et al. [42] found an association between impaired muscle function and 6-year hip fractures. Overall, sarcopenic obesity has negative impacts on bone and predisposes worse bone microarchitecture compared to obese people of the same BMI without sarcopenia [3]. The mechanism leading to impaired muscle dysfunction in obesity remains unclear. However, it has been demonstrated that pro-inflammatory cytokines present in muscles (e.g., TNFα and IL-6), which are increased in obesity, can be reduced by exercise [43].

**Figure 1:**
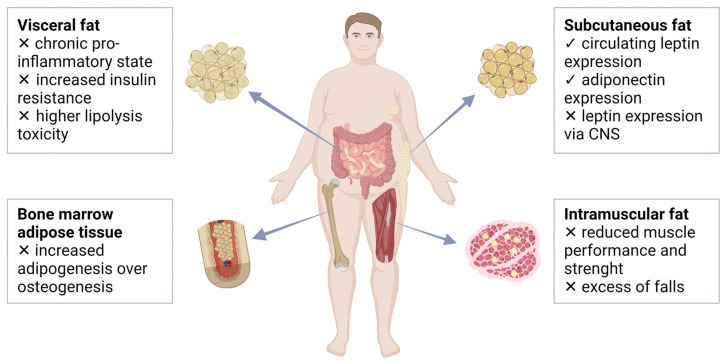
Positive (✓) and negative (✗) impacts of fat depot actions in relation to bone health in obesity (created with BioRender.com; accessed on 5 March 2024). Abbreviations: CNS—central nervous system.

Obesity and T2DM significantly influence microstructural changes in bone marrow and contribute to the disruption of bone homeostasis, resulting in a higher risk of fragility fractures [28]. An altered cellular landscape and molecular networks within the bone microenvironment lead to an increase in bone marrow adipose tissue volume. It is induced by an increase in the size and/or number of adipocytes and may cause a restriction of space for other cells that are required for normal skeletal homeostasis, such as bone marrow stromal cells, hematopoietic cells, or osteoblastic cells [44]. Moreover, factors that are secreted by bone marrow adipocytes (e.g., adipokines, pro-inflammatory and immunoregulatory cytokines, and receptor activator of nuclear factor kappa beta ligand (RANKL)) may also contribute to altered skeletal homeostasis after bone marrow adipose tissue expansion [9,45]. In addition, accumulation of senescent cells and elevated levels of oxidative stress in the bone microenvironment in obesity and T2DM are among factors that may participate in bone marrow stromal cell dysfunction and cause a shift in bone marrow stromal cell differentiation phenotype that favors adipogenesis over osteogenesis [2]. The expansion of bone marrow adipose tissue was observed in bone biopsies from obese elderly men [46]. According to Tencerova et al. [47], bone marrow stromal cells obtained from obese men showed an increased adipocyte differentiation and accelerated senescence phenotype that might contribute to skeletal fragility in obesity. Interestingly, bone marrow adipose tissue was elevated in women with anorexia nervosa, who have very low levels of visceral and subcutaneous fats. Moreover, bone marrow fat was inversely correlated with BMD [48]. This suggests that while other fat stores tend to be highly correlated, bone marrow adipose tissue has a different pattern, suggesting separate control mechanisms [49]. As bone marrow fat increases, saturated lipids appear to elevate over unsaturated lipids [50]. It is known that magnetic resonance spectroscopy (MRS) of bone marrow lipid profiles from peripheral skeletal sites may be a promising tool for identifying individuals with osteoporosis or at risk of developing osteoporosis [51,52]. Figure 1 shows both the positive and negative effects of fat deposits in relation to bone health in obesity.

## 3. Biologically Active Molecules Responsible for Impaired Bone Quality in Obesity and T2DM

Adipose tissue is considered an endocrine organ with a decisive role in energy homeostasis. Various molecular pathways by which adipose tissue communicates with bone tissue have been proposed. This interplay is active, dynamic, and involves multiple factors such as hormones (e.g., leptin, adiponectin, and resistin), pro-inflammatory cytokines (e.g., TNF-α, IL-6, and CRP), and vitamin D. In addition, bone tissue influences metabolic parameters, including body weight control through bone-derived factors (e.g., osteocalcin and osteopontin) [53]. In general, biochemical markers of bone turnover are reduced in obese individuals [54]. However, López-Gómez et al. [55] reported age-related differences in the levels of bone turnover markers in postmenopausal obese women, where markers of bone formation were reduced especially at younger ages and markers of bone resorption were increased at older ages.

Leptin, which is elevated in obese individuals, has a dual effect on bone. It acts on the hypothalamus to activate the sympathetic nervous system and inhibit bone formation (negative impact), but it also acts directly on osteoblasts to stimulate bone formation (positive impact). The negative effect seems to outweigh the positive one [56,57]. Adiponectin is reduced in obese subjects. It stimulates osteoblastogenesis and suppresses osteoclastogenesis (both osteoblasts and osteoclasts express adiponectin receptors). However, adiponectin concentrations are inversely proportional to pro-inflammatory cytokine levels (e.g., CRP, TNF-α, and IL-6), which serve as potent inhibitors of adiponectin expression [53,58]. Resistin, which is increased in obese patients, has a controversial effect on bone. It appears to promote osteoblast proliferation but also supports osteoclast proliferation and the release of pro-inflammatory cytokines [59,60]. In obese individuals, serum levels of vitamin D are significantly lower compared to non-obese subjects. However, obese individuals have higher BMD, suggesting that a low vitamin D level may not be related to adverse bone outcomes [61,62]. Osteocalcin (OC), which is lower in obese subjects and is considered a bone formation marker, improves glucose intolerance, obesity, and insulin expression by controlling gene expression in β-cells and adipocytes. In addition, it stimulates adiponectin secretion [11,63]. Osteopontin is elevated in obese individuals and is involved in physiological and pathological bone mineralization. Its expression is strongly upregulated in adipose tissue in obesity. Antibody-mediated neutralization of osteopontin action was found to significantly reduce insulin resistance in obesity [64,65].

Both obesity and T2DM are associated with oxidative stress and inflammation. Oxidative stress is induced by reactive oxygen species (ROS), which increase with aging or the onset of an inflammatory state and can harmfully affect bone homeostasis. There is evidence that ROS (e.g., superoxide and hydrogen peroxide) are important for a wide variety of different signaling pathways, including the regulation of mitogen-activated protein kinases (MAPKs), transcription factors, and intracellular Ca^2+^ levels [66]. In general, ROS are decisive components that regulate osteoclast formation in a process mediated by the interaction between RANKL and receptor activator of nuclear factor kappa beta (RANK). Osteoprotegerin (OPG), which is a soluble decoy receptor for RANKL, prevents binding of RANKL to RANK. Therefore, the RANK/RANKL/OPG pathway is a key mediator of osteoclastogenesis [67,68]. Moreover, ROS induce apoptosis of osteoblasts and osteocytes by activating many signaling pathways. In this process, MAPKs (e.g., JNK and ERK) are involved. ROS also reduce osteoblast activity and differentiation, and thus, mineralization and osteogenesis [69,70]. In this context, it is important to mention that the Wnt/β-catenin signaling pathway is essential for bone formation [71]. β-catenin is a crucial transcription co-activator that regulates transcription of several target genes, including runt-related transcription factor 2 (Runx2), which is responsible for osteoblast differentiation [72].

Obesity is also related to chronic low-grade inflammation resulting in insulin resistance and, ultimately, T2DM [73]. It is more pronounced in central and visceral adiposity and characterized by higher levels of pro-inflammatory markers (e.g., IL-6, TNF-α, and CRP) and pro-resorptive factors (e.g., RANKL and tartrate-resistant acid phosphatase 5b (TRAP5b)). Obesity is also linked to the activation of peroxisome proliferator-activated receptor γ (PPAR-γ), the nuclear factor kappa light chain enhancer of activated B cells (NF-κB), and CCAAT/enhancer-binding protein α (C/EBPα) pathways [53]. Activation of PPAR-γ promotes the differentiation of mesenchymal stem cells into adipocytes rather than osteoblasts [74]. NF-κB activation is required for osteoclast differentiation [75]. C/EBPα is critical for osteoclast differentiation and activity [76]. Moreover, adipokines (e.g., adiponectin, leptin, and resistin) produced in adipocytes have an inverse relationship with fat mass [77] and variably influence bone mass [78].

**Figure 2:**
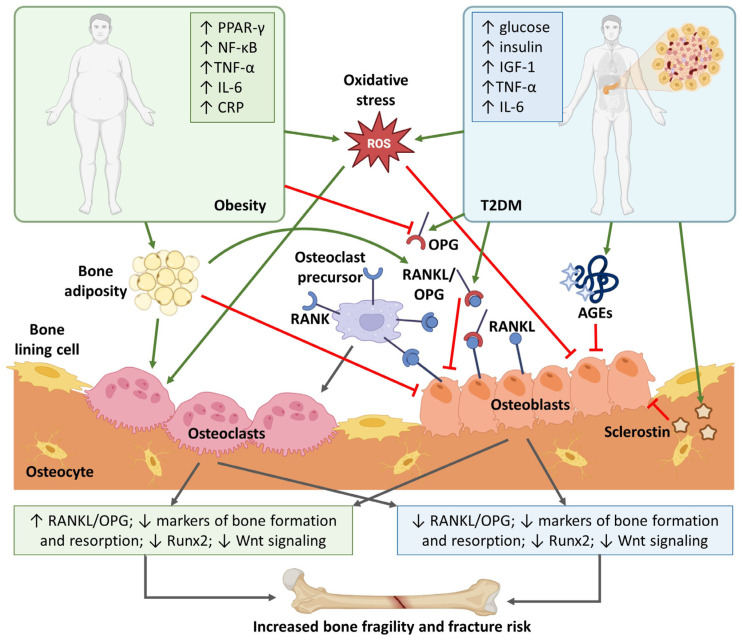
Schematic representation of the action of individual molecules responsible for bone fragility and fracture risk in obesity and T2DM (created with BioRender.com; accessed on 5 March 2024). Abbreviations: AGEs, advanced glycation end products; CRP, C-reactive protein; IGF-1, insulin-like growth factor 1; IL-6, interleukin-6; NF-κB, nuclear factor kappa light chain enhancer of activated B cells; OPG, osteoprotegerin; PPAR-γ, peroxisome proliferator-activated receptor gamma; RANK, receptor activator of NF-κB; RANKL, receptor activator of NF-κB ligand; ROS, reactive oxygen species; Runx2, runt-related transcription factor 2; T2DM, type 2 diabetes mellitus; TNF-α, tumor necrosis factor alpha.

There are some similarities between impaired bone quality in obesity and T2DM, but T2DM has more deleterious effects on the skeleton. Many studies suggest that due to chronic hyperglycemia, the formation of ROS may be associated with collagen glycation, which is an important factor [4,5]. Overall, bone damage in T2DM can be caused by multiple mechanisms: accumulation of advanced glycation end products (AGEs) [79], increased levels of pro-inflammatory cytokines (e.g., IL-6, TNF-α) [80], sclerostin [81], leptin [80], lower levels of OC [11,82]), procollagen I N-terminal propeptide (P1NP) [83,84], parathyroid hormone (PTH) [85], and impairment of osteoblastogenesis [86]. The status of low bone turnover in T2DM is also reflected in lower levels of bone resorption markers (e.g., TRAP5b and C-telopeptide of type I collagen (CTx)) [82,85]. In addition to elevated oxidative stress, hyperglycemia is related to decreased osteoblast function, inhibited bone mineralization, enhanced adipogenesis, and downregulation of vitamin D receptors. All aforementioned conditions lead to a disturbance of bone metabolism, worse bone quality, higher fracture risk [47,87,88,89,90], and osteoporosis [91]. According to Zhang et al. [71], the insulin-like growth factor (IGF)-1/β-catenin signaling axis plays an essential role in the pathogenesis of osteoporosis in T2DM, as IGF-1 is involved in both bone and glucose metabolism and IGF-1 signaling regulates the Wnt/β-catenin signaling pathway. Figure 2 illustrates the involvement of individual molecules, responsible for bone damage, in obesity and T2DM. Given that obesity is a risk factor for T2DM, it would be difficult to separate the impact of obesity on bone health from that of T2DM. Therefore, this figure represents their simultaneous effect on bone fragility and fracture risk.

## 4. Relationships between Obesity and Osteoporosis

In recent years, complicated interaction between obesity and osteoporosis has attracted considerable attention, as adiposity has been shown to have a fundamental function in bone metabolism [92]. Consequently, there has been an increasing trend of studies to assess their relationship, which may differ mainly depending on the indicators of obesity used (e.g., BMI, WC, and waist-to-hip ratio (WHR)) [93,94] and/or assessment methods for BMD (e.g., dual energy X-ray absorptiometry (DEXA), high-resolution peripheral quantitative computed tomography (HR-pQCT), trabecular bone score (TBS), and lumbar spine BMD/BMI ratio (LS BMD/BMI ratio)) [19,60].

In general, fat distribution and body composition can vary significantly between individuals with identical BMIs due to different percentages of fat mass or muscle mass [3]. Therefore, WHR or fat mass index (FMI) may serve as a better screening tool to predict body fat percentage [95,96]. According to Ross et al. [97], a combination of WC and BMI can identify the highest-risk phenotype of obesity much better than each of these indicators individually. In this context, Khan et al. [98] reported that WHR had the most robust association with adiposity-mortality risk compared to BMI and FMI; therefore, it may serve as the most appropriate obesity indicator.

Since osteoporosis is no longer considered solely a disease of low BMD, other factors including bone turnover, microarchitecture, and geometry that contribute to bone strength and/or are linked to osteoporotic bone must be taken into account. BMD is a useful diagnostic tool; however, it reflects only one component of bone strength [99]. This reality is also confirmed by several studies indicating that fragility fractures are identified even in individuals with a T-score > −2.5 SD [100,101,102,103,104,105]. Generally, osteoporosis is characterized by a deficit in either bone quantity, bone quality, or both [99]. Bone quantity can be measured using DEXA or quantitative ultrasound (QUS). However, these instrumental methods cannot analyze the quality of bones. In general, HR-pQCT, TBS, and LS BMD/BMI ratio are used for this purpose. The latter assessment methods are, thus, more reliable and better than DEXA or QUS in evaluating complex bone health [60].

In obese individuals, BMD is known to be increased and/or unchanged, but elevated BMD and soft tissue thickness may introduce precision error in DEXA measurement through assumptions about abdominal thickness and beam stiffening effects [4,106]. In this context, Blake et al. [107] mentioned that individuals with a lower spine T-score had a significantly higher vertebral marrow fat content. Therefore, the ability of DEXA scans to determine fracture risk may be explained in part by the effect of increased vertebral marrow fat on BMD. According to Ruosi et al. [74], BMD was normal but the spine deformity index (SDI) showed reduced bone quality, and vertebral compression fractures were observed in 87.5% of obese patients. Romagnoli et al. [108] reported that obesity negatively affected TBS, despite unchanged BMD. TBS was inversely related to BMI, indicating that increases in BMI adversely influenced bone quality. Moreover, the impact of WC on TBS was more significant than that of BMI. Eller-Vainicher et al. [109] stated that TBS and LS BMD/BMI ratios were nearly always reduced more than BMD in obese individuals. Since several studies have revealed a greater site-dependent fracture risk in obese individuals [110,111,112], TBS and LS BMD/BMI ratio may provide a better perspective for determining the risk of fragility fractures in obese subjects.

In the past, it was assumed that individuals most at risk of fractures were non-obese women. A lower BMI has been suggested to elevate the risk of osteoporosis [113,114]. This initial belief was mainly supported by a positive relationship between BMI and BMD [113,115]. Cherif et al. [116] pointed to an overall high BMD in postmenopausal obese women. Hammoud et al. [117] found that the severity of obesity did not affect BMD values in premenopausal obese women. A meta-analysis by Qiao et al. [118] revealed a positive association between obesity (determined based on BMI) and femoral neck BMD, and between obesity and lumbar spine BMD, in adults. Therefore, a negative correlation between obesity and osteoporosis was established. Similarly, in a meta-analysis by Liu et al. [119], general obesity (measured by BMI) was associated with lower odds of developing osteoporosis. However, central obesity (measured by WC) showed no association with osteoporosis. On the contrary, Pamganamamula et al. [120] in their meta-analysis found that patients in the obese BMI category had a higher incidence of osteoporosis compared to other relevant groups (overweight, normal, and underweight), suggesting that a higher BMI does not reflect a protective role against osteoporosis (BMD was determined in the lumbar spine and femoral neck).

**Figure 3:**
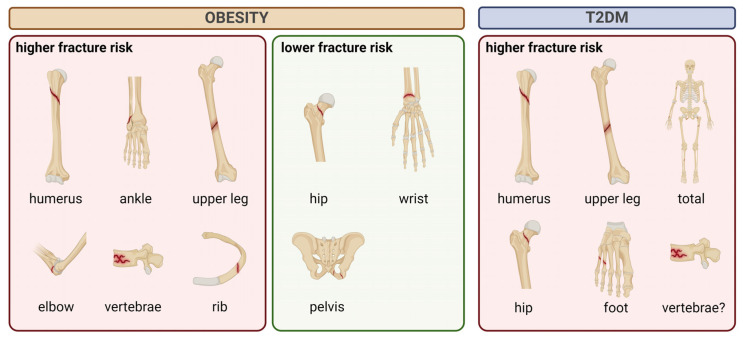
Schematic illustration of higher and lower site-specific fracture risk in obesity and T2DM (created with BioRender.com; accessed on 5 March 2024). Abbreviations: T2DM, type 2 diabetes mellitus.

Most research studies suggest that the link between obesity and fracture risk is more complex than previously thought. For example, it may be modified by the interaction between body weight and BMD; obesity has a different effect on fracture risk at different sites of the skeleton; and the relationship between obesity and fracture risk is dependent on both age and sex [121,122,123]. According to Prieto-Alhambra et al. [111], obesity is protective against hip and pelvic fractures in postmenopausal women, but is related to an almost 30% increased risk of proximal humerus fractures. The protective impact of obesity against hip fractures, but a higher risk of humerus fractures, was also demonstrated by Gnudi et al. [124]. A meta-analysis of Tang et al. [125] confirmed a negative association between obesity and hip fracture risk in adults. On the other hand, Sadeghi et al. [112] found a positive relationship between these two indicators (obesity and hip fractures). According to Compston et al. [110], the risk of wrist and hip fractures was reduced, while the risk of ankle and upper leg fractures was elevated in postmenopausal obese women. The authors found a 27% prevalence of the last two fractures mentioned in older obese women, who were also more likely to have experienced early menopause and reported two or more falls (obese individuals most often fall backwards or sidewards, while non-obese subjects most often fall forwards) in the past year. An increased risk of ankle and upper arm fractures and a lower risk of wrist fractures in obese patients were also observed by Ong et al. [126]. According to Tanaka et al. [127], vertebral fractures occurred more frequently in postmenopausal obese women, while femoral neck fractures and long-bone fractures were less common. Similarly, Liu et al. [128] established an elevated risk of vertebral fractures in postmenopausal obese women, but obesity served as a protective factor for pelvic fractures. Kaze et al. [129] found that obesity reduced the risk of vertebral fractures in men but not in women, suggesting possible gender-related differences. Johansson et al. [130] in their meta-analysis revealed that obesity was a risk factor for humerus and elbow fractures in adult women, while low BMI was a risk factor for hip fractures and all osteoporotic fractures. In a meta-analysis by Turcotte et al. [131], postmenopausal obese women were found to have a 60% higher risk of ankle fracture than non-obese women. On the contrary, risks of hip and wrist fractures were reduced by 25% and 15%, respectively, in obese women. In older men, obesity was associated with a reduced risk of clinical fracture of the spine, hip, pelvis, and wrist, and an increased risk of multiple rib fractures [132]. In this case, obesity-induced hypogonadism may contribute to bone fragility, as obese men have been shown to have low testosterone levels [133]. Surprisingly, Premaor et al. [134] demonstrated an unaccountably high (28%) prevalence of obesity in postmenopausal women with a low-trauma fracture, most of whom had normal BMD (measured by DEXA). Figure 3 shows higher and lower site-specific fracture risk in obese adults.

It is widely known that bone turnover and bone resorption are reduced in obese individuals. Therefore, the question of whether anti-resorptive treatment is effective in preventing fractures in obesity is relevant [135]. A study by McClung et al. [136] demonstrated that denosumab reduced the risk of vertebral fractures in both obese and non-obese postmenopausal women independent of BMI, although a decrease in non-vertebral fractures was identified only in those without obesity. Treatment with bisphosphonates (e.g., zoledronic acid) caused a greater reduction in vertebral fracture risk in postmenopausal women with BMI ≥ 25 kg/m^2^ versus those with BMI < 25 kg/m^2^, but had no significant effect on non-vertebral fractures [137]. These data indicate that higher doses of anti-resorptive drugs may be required in obese patients to maintain effectiveness against non-vertebral fractures [138]. In addition, obese women with a fracture were found to be more frequently undertreated than non-obese women (27% vs. 41%) due to higher BMD and specific “non-osteoporotic” fractures (e.g., ankle) [110]. In any case, further research should be conducted in this area.

## 5. Relationships between T2DM and Osteoporosis

Both T2DM and osteoporosis are influenced by lifestyle changes and aging and quite often coexist, especially in the elderly. There is a complex pathophysiological interaction between the two diseases: T2DM directly affects bone strength and bone metabolism, some antidiabetic medications influence bone metabolism, and there is a link between T2DM-related complications and the risk of falls and subsequent fractures [139,140]. A meta-analysis of Si et al. [141] revealed 44.8% and 37.0% prevalence of osteoporosis in diabetic women and men, respectively. According to Wang et al. [142], the incidence of osteoporosis was 20.6% in older women with T2DM and 5.0% in older men with T2DM. A current meta-analysis by Liu et al. [91] determined a 27.67% prevalence of osteoporosis in T2DM patients worldwide, which could be a reason to implement osteoporosis control in diabetic patients.

Several studies have shown that overall fracture risk is increased in subjects with T2DM, being higher with poor glycemic control, longer T2DM duration, and diabetic complications [143,144]. Interestingly, high variability in fasting glucose was consistent with an elevated risk of hip fracture [145]. Conversely, patients with impaired glucose tolerance were not at increased risk of fracture and may in fact have a lower risk [146]. It is hypothesized that it could be related to high BMI and insulin resistance, which are often determined in individuals with impaired glucose tolerance [147]. In general, higher urinary Ca excretion, functional hypoparathyroidism, central hypogonadism, and alterations in IGF-1 and vitamin D metabolism have been implicated in raising the risk of fractures [148,149].

Despite higher and/or unchanged BMD, several authors have reported an increased risk of hip, upper leg, foot, humerus, and total fractures in older patients with T2DM [148,150,151,152,153,154,155,156]. Although both women and men with T2DM appear to have an elevated risk of hip and non-vertebral fractures [151,157], a cohort study by Napoli et al. [158] showed no difference in the incidence of vertebral fractures between elderly men with and without T2DM. Similarly, Dytfeld and Michalak [159] revealed that T2DM does not increase the risk of vertebral fractures in postmenopausal women, but there were relatively few data on vertebral fractures. On the contrary, Viégas et al. [160] found a high prevalence (23%) of vertebral fractures among postmenopausal women with T2DM. A meta-analysis of Moayeri et al. [156] also showed a positive association between T2DM and vertebral fractures in adults. Moreover, fracture incidence increased with age and was higher in men with T2DM compared to women with T2DM. Figure 3 illustrates sites at higher fracture risk in T2DM adults.

Prolonged fracture healing has also been linked to T2DM. Overall, T2DM significantly increases the rate of specific unfavorable effects in surgically treated lower limb fractures, including nonunion, deep infection, and reoperation. In addition, a greater risk of nonunion in fractures below the knee has been reported [161,162]. Post-fracture patients with T2DM generally have higher mortality, develop more complications, and recover worse than individuals without T2DM [4].

In older patients with T2DM, a higher risk of falls has been recorded, partly due to elevated bone fragility, hypoglycemic events, and obesity, but also due to T2DM-related disorders. Peripheral neuropathy may be related to poor balance and localized bone loss, which may further elevate the risk of fall-related fractures, particularly at the foot and ankle. Diabetic retinopathy can further compromise the patient’s safety and lead to gait instability. Patients with diabetic nephropathy, which is associated with disorders of bone and mineral metabolism, are prone to fractures due to renal osteodystrophy and also to falls. Cardiovascular diseases (e.g., cardiac arrhythmia, stroke, and atherosclerosis) are considered common comorbidities in T2DM and can also increase the risk of collapse, falls, and fractures [134,150,163,164,165,166].

Bone strength and fracture risk are determined by bone quantity and quality. In T2DM patients, BMD does not seem to reflect the risk of fractures. Impaired bone quality contributes to bone fragility and elevates fracture risk independently of BMD [152]. Overall, adults with T2DM have a lower TBS compared to those with normal glycemia. In addition, TBS predicts osteoporotic fractures and captures a greater proportion of the fracture risk than BMD [167,168]. Using HR-pQCT, lower cortical volumetric (v)BMD and increased cortical porosity linked to reduced bone strength, and a greater fracture incidence, were reported in elderly subjects with T2DM [169,170]. In T2DM patients, cortical resistance (an index of bone material strength) was lower and this parameter decreased with worsening glycemic control. Accordingly, higher contents of total mineral and AGEs were noted in subjects with T2DM, consistent with reduced bone turnover [171]. Bone turnover in T2DM is indeed low with decreased bone formation and, to a lesser extent, bone resorption [172,173].

Pharmacotherapy of T2DM can also modulate the risk of fractures. Metformin, sulfonylureas, glucagon-like peptide-1 receptor agonists, and dipeptidyl peptidase-4 inhibitors have neutral or protective associations with fracture risk [4,139]. Thiazolidinediones (TZDs) and the sodium–glucose cotransporter 2 (SGLT2) inhibitor, canagliflozin, are known to elevate fracture risk [174,175]. Therefore, TZDs should not be used and SGLT2 inhibitors should be used with caution in T2DM patients at high risk of fracture. Insulin therapy is the preferred method to achieve glycemic control in T2DM subjects with fractures. However, it should be used carefully to avoid hypoglycemia and elevated fracture risk [4,139]. Pharmacological treatment of osteoporosis generally reduces the risk of fractures in patients with T2DM. Bisphosphonates (e.g., alendronate) and selective estrogen receptor modulators (e.g., raloxifene) have similar efficacy against vertebral fractures in individuals with and without T2DM [176,177]. Denosumab significantly reduces vertebral fracture risk in patients with T2DM and osteoporosis [178]. Teriparatide significantly lowers non-vertebral fracture risk in subjects with and without T2DM [176,179]. According to Eastell et al. [180], bisphosphonates and most other anti-resorptive drugs (e.g., denosumab, selective estrogen receptor modulators, and odanacatib) have similar efficacy in reducing fracture risk in individuals with and without T2DM.

## 6. MicroRNAs as Biomarkers in Obesity, T2DM, and Osteoporosis

From the text mentioned above, it appears that diagnostic tools for identifying patients at risk of fracture are insufficient in both obesity and T2DM. Therefore, it is necessary to specify novel biomarkers that will help determine bone fragility in these diseases [181]. MicroRNAs (miRNAs) are currently receiving considerable attention as biomarkers for various diseases, including obesity, T2DM, and osteoporosis [182]. Based on several preclinical studies, microRNAs with functional roles in the induction of bone/fat switch and bone defects may become important therapeutic targets [183]. Saferding et al. [184] identified miR-146a as a molecular checkpoint controlling age-related bone loss by limiting bone anabolic pathways and promoting bone marrow adiposity. In postmenopausal women and men with fragility fractures, miR-146a levels were increased. Therefore, targeting miR-146a may be an effective means of treating bone loss in osteoporosis. Furthermore, miR-21 and miR-133a were found to be related to BMD in postmenopausal osteoporotic women [185]. Jordan et al. [186] demonstrated that miR-143 was a positive regulator of human adipocyte differentiation acting through ERK5 signaling. Studies by Lee et al. [187] and Yu et al. [188] pointed out that miR-27a and miR-130a inhibited adipocyte differentiation via PPAR-γ downregulation. According to Villard et al. [189], miR-140-5p, miR-142-3p, and miR-222 were upregulated and miR-21-5p, miR-103-5p, miR-125-5p, and miR-221-3p were downregulated in obese patients, while both miR-142-3p and miR-222 were commonly upregulated in obese and T2DM patients. In addition, in silico analysis of targeted genes and pathways indicated the potential role of miR-142-3p and miR-222 in the metabolic properties of both obese and T2DM patients. Brovkina et al. [190] found that miR-23b-3p and miR197-3p were increased in obese patients. Moreover, miR-99b, miR-125a-5p, miR125b-5p, miR-204-5p, and miR320a were upregulated in obese patients with T2DM. Interestingly, miR-328-3p and miR-328 were downregulated and upregulated in obesity, respectively, and miR-328 was downregulated in sarcopenia. Plasma miR-215 was upregulated in both obesity and sarcopenia, and miR-215-5p was also upregulated in obesity [191,192].

The findings of Heilmeier et al. [193] revealed miR-550a-5p and miR-382-3p as potential candidates for indicating fragility status in postmenopausal women with T2DM, while miR-188-3p could potentially be used as an indicator of osteoporosis-related fractures. According to Jiang et al. [194], the miR-222 inhibitor could potentially be used to accelerate bone healing in fractured STZ-induced diabetic rat model and, therefore, may be considered as a therapeutic target. Zhang et al. [195] reported that miR-205 was involved in osteogenic/adipogenic differentiation of bone marrow mesenchymal stem cells by targeted inhibition of the expression of Runx2 in elderly female mice with T2DM and osteoporosis. Therefore, miR-205/Runx2 may be a target for the treatment of patients suffering from both T2DM and osteoporosis.

## 7. Conclusions

Obesity, T2DM, and osteoporosis are serious diseases with increased morbidity; mortality; and various secondary complications, which often occur together in older individuals. Despite higher and/or unchanged BMD, subjects with obesity and T2DM have poor bone quality and an increased risk of fragility fractures at specific sites.

In general, obesity is a protective factor against hip, pelvis, and wrist fractures in older patients. On the contrary, an increased risk of humerus, ankle, upper leg, elbow, vertebrae, and rib fractures is determined in obese subjects. Overall, the pathophysiology of aforementioned fractures is not fully understood, but an excess of falls, various patterns of falling, and unfavorable impacts of adipose tissue on bone tissue can contribute to this. Bisphosphonates and denosumab significantly reduced vertebral fracture risk in postmenopausal obese women. It is expected that higher doses of anti-resorptive drugs may be required in obese subjects to maintain effectiveness against non-vertebral fractures. In older patients with T2DM, a greater risk of hip, upper leg, foot, humerus, and total fractures was established. In addition, post-fracture patients experience an excess of falls, prolonged fracture healing, and elevated mortality. Pharmacotherapy can also modulate the risk of fracture in T2DM. In general, TZDs should not be used and SGLT2 inhibitors should be used with caution in T2DM patients at high risk of fracture due to an even increased fracture risk. Bisphosphonates, selective estrogen receptor modulators, and denosumab significantly reduced vertebral fracture risk in patients with T2DM. Teriparatide significantly lowered non-vertebral fractures in T2DM subjects.

It is important to be aware of elevated fracture risk and osteoporosis in patients with obesity and T2DM, as they are currently considered low-risk and tend to be underdiagnosed and undertreated due to the lack of bone quality assessment by DEXA or QUS. Therefore, implementation of better diagnostic tools to predict body fat percentage and assess comprehensive bone health including fragility fracture risk in obese and T2DM subjects (e.g., WC, WHR, MRS, HR-pQCT, TBS; LS BMD/BMI ratio, and miRNAs) can be considered an important clinical and future priority that could improve fracture prevention in this group of patients.
